# An atypical finding on serum immunofixation: a case report

**DOI:** 10.1093/labmed/lmaf060

**Published:** 2025-10-09

**Authors:** Ameerah Davids, David F Keren, Annalise E Zemlin, Fatima B Fazel, David L Murray, Ernest Musekwa, Marizna Korf

**Affiliations:** Division of Chemical Pathology, Department of Pathology, Faculty of Medicine and Health Sciences, Stellenbosch University, Cape Town, South Africa; Division of Chemical Pathology, Department of Pathology, National Health Laboratory Service, Tygerberg Hospital, Cape Town, South Africa; Department of Pathology, The University of Michigan, Ann Arbor, MI, United States; Division of Chemical Pathology, Department of Pathology, Faculty of Medicine and Health Sciences, Stellenbosch University, Cape Town, South Africa; Division of Chemical Pathology, Department of Pathology, National Health Laboratory Service, Tygerberg Hospital, Cape Town, South Africa; Division of Clinical Haematology, Department of Internal Medicine, Faculty of Medicine and Health Sciences, Stellenbosch University and Tygerberg Hospital, Cape Town, South Africa; Department of Laboratory Medicine and Pathology, Mayo Clinic, Rochester, MN, United States; Division of Haematological Pathology, Department of Pathology, Faculty of Medicine and Health Sciences, Stellenbosch University and National Health Laboratory Services, Cape Town, South Africa; Division of Chemical Pathology, Department of Pathology, Faculty of Health Sciences, University of Cape Town, Cape Town, South Africa; Division of Chemical Pathology, National Health Laboratory Service, Groote Schuur Hospital, Cape Town, South Africa

**Keywords:** multiple myeloma, Mass-Fix, serum free light chains, serum immunofixation

## Abstract

**Introduction:**

Multiple myeloma (MM) is characterized by the abnormal proliferation of plasma cells, resulting in the overproduction of distinctive monoclonal proteins (M-protein). Suspected MM necessitates screening for M-protein through a combination of serum protein electrophoresis, serum immunofixation (SIFE), and serum free light chain (SFLC) determination. An M-protein appears as a relatively restricted band on agarose gel, where migration in ɑ-2 is rare.

**Methods:**

A 55-year-old man with pulmonary tuberculosis presented with severe lower back pain. On examination, he appeared chronically ill, with conjunctival pallor. X-rays revealed vertebral compression fractures. The full blood count confirmed anemia; however, serum calcium and creatinine levels did not meet myeloma-defining event criteria.

**Results:**

The serum protein electrophoresis revealed hypogammaglobulinemia, with the SIFE demonstrating unusual unrestricted κ staining in the ɑ-2 region. A markedly elevated κ SFLC and κ:λ ratio were found. Bone marrow examination demonstrated approximately 90% plasmacytosis. Urine immunofixation revealed a small, restricted κ band disproportionate to the κ SFLC. Notably, matrix-assisted laser desorption/ionization time-of-flight mass spectrometry identified only polyclonal κ SFLC.

**Discussion:**

Given the absence of a discernible M-protein on SIFE, a small κ restriction on urine immunofixation, and a polyclonal increase in κ SFLCs, the patient’s condition is being managed as an oligosecretory MM.

## Introduction

Multiple myeloma (MM) is characterized by the proliferation of a malignant plasma cell clone secreting intact monoclonal immunoglobulins or free light chains, also known as M-proteins. These proteins drive the clinical manifestations of target organ damage in MM, known as the CRAB criteria (hypercalcemia, renal impairment, anemia, and osteolytic bone lesions), and are crucial in distinguishing MM from other plasma cell disorders.^1,2^ When attributable to a plasma cell disorder, CRAB features are considered myeloma-defining events (MDEs).^3,4^ In 2014, the International Myeloma Working Group expanded the diagnostic criteria by incorporating myeloma-defining biomarkers, enabling earlier diagnosis and treatment initiation: 60% or more bone marrow plasma cell infiltration, an involved to uninvolved serum free light chain (SFLC) ratio of 100 or higher, or more than 1 focal lesion larger than 5 mm on magnetic resonance imaging.^3,5^

When clinically suspected, it is recommended to screen for the presence of an M-protein using a combination of serum protein electrophoresis (SPE), serum immunofixation (SIFE), and SFLC determination.^6^ The M-protein appears as a relatively restricted band on agarose gel electrophoresis and immunofixation.^7^

This case describes a patient with an initial diagnosis of κ light chain MM due to a clinically significantly elevated κ SFLC at diagnosis, with a plasmacytosis of 90% on bone marrow aspirate and trephine (BMAT) biopsy in the absence of detectable M-protein on SIFE. Further analysis with a matrix-assisted laser desorption/ionization time-of-flight mass spectrometry method (Mass-Fix), however, revealed only a polyclonal increase in κ SFLC, complicating the diagnostic interpretation of the biochemistry results.

## Methods

### Case report

A 55-year-old man with a history of pulmonary tuberculosis (TB) diagnosed in 2006 and complicated by post-TB structural lung disease, was diagnosed with recurrent sputum-positive, rifampicin-sensitive TB in August 2022. One month after starting TB treatment, he presented again with worsening lower back pain and was wheelchair-bound due to the severity of the pain. He had no constitutional symptoms (fever, night sweats, or recent weight loss). On examination, he appeared chronically ill, with conjunctival pallor and pedal edema. He weighed 45 kg (body mass index, 17.6 kg/m^2^), and diffuse lumbar spine tenderness was noted. At the time, he was on anti-TB therapy (rifampin, isoniazid, pyrazinamide, ethambutol [Rifafour (Sanofi-Aventis South Africa)]), pyridoxine, and analgesics.

**Figure 1.**
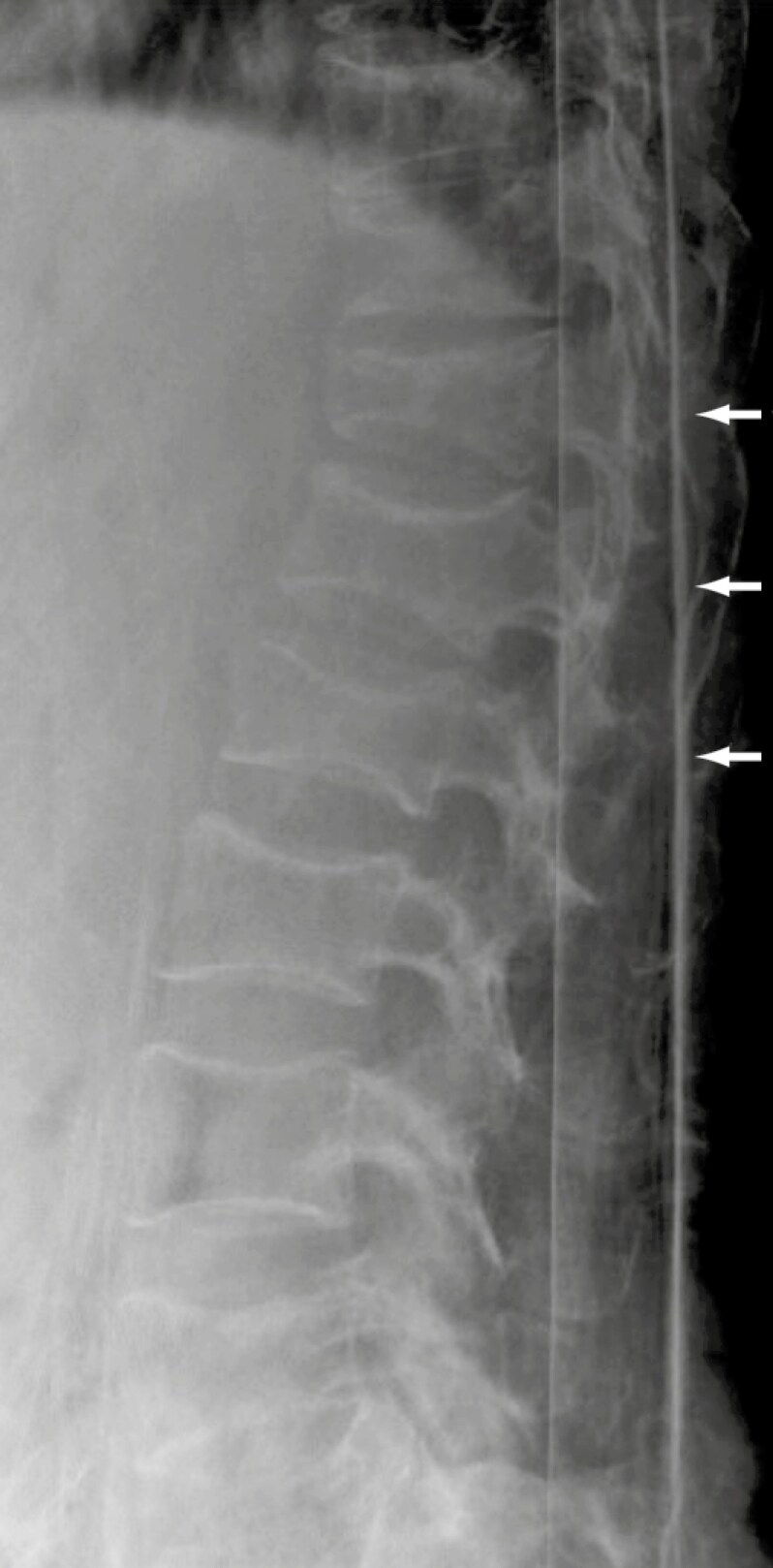
Lateral thoracolumbar spine x-ray showing multilevel wedge compression fractures.

A lateral spinal x-ray revealed multiple thoracolumbar vertebral compression fractures, with sparing of the disc spaces and no overt lytic lesions (Figure 1). A chest x-ray showed right middle lobe opacification and left upper lobe fibrocavitatory changes, consistent with both active and past TB. Computerized tomography (CT) scans of the chest confirmed right middle lobe consolidation, a fibrocavitatory lesion in the left lung apex, and bilateral upper lobe fibrosis, again suggestive of active and past TB. Orthopedics recommended a prolonged course of anti-TB therapy due to suspected spinal involvement. Given the patient’s symptoms, anemia, and concerning spinal imaging, SPE and SFLCs were ordered to screen for MM. The patient was referred to a step-down facility for rehabilitation and pain management.

**Figure 2.**
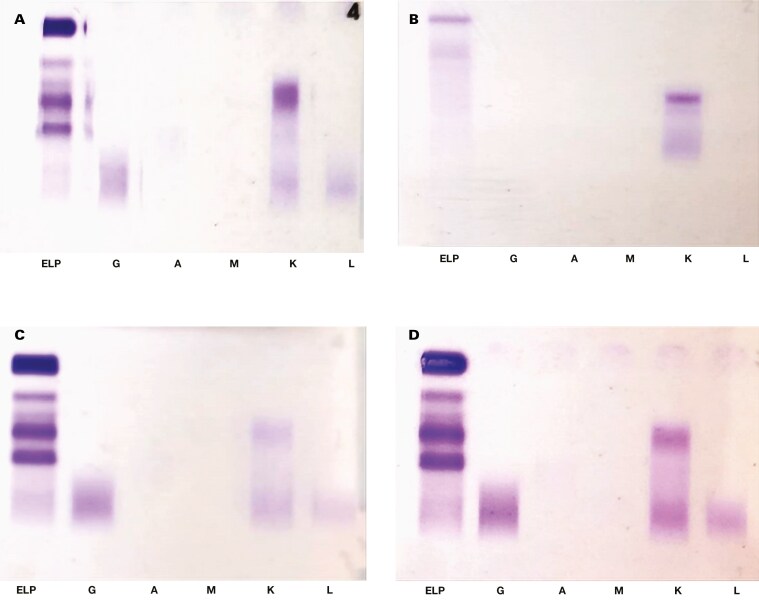
Immunofixations. (**A**) Serum immunofixation (September 2022); (**B**) urine immunofixation; (**C**) serum immunofixation (February 2023); (**D**) serum immunofixation after pre-treatment with beta-mercaptoethanol (February 2023)

On admission in September 2022, mild early-stage renal impairment and a normal total serum calcium concentration were noted (Table 1). Full blood count showed macrocytic anemia and mild thrombocytopenia, with a normal nutritional screen. The peripheral smear confirmed true thrombocytopenia, round macrocytes, and no circulating plasma cells. The SPE revealed hypogammaglobulinemia of 3 g/L (reference interval, 6-12 g/L), with no observable area of restriction. A reflex SIFE showed abnormal κ staining in the ɑ-2 region, but it was not restricted and therefore reported as negative (Figure 2A). The SFLC analysis demonstrated markedly elevated κ SFLCs and an increased κ:λ ratio, raising concern for a plasma cell disorder.

**Table 1.** Laboratory Investigations

Analyte	September 24, 2022 admission (index presentation)	December 6, 2022 local clinic consultation	December 21, 2022, hematology index consultation (cyclophosphamide and dexamethasone initiated)	September 26, 2023 hematology follow-up (cyclophosphamide and dexamethasone continued)	November 5, 2024 (lenalidomide initiated in combination with cyclophosphamide and dexamethasone)	February 3, 2025 hematology follow-up	Reference interval
Urea, mmol/L	9.1	18.0	13.7	6.7	7.0	5.3	2.1-7.1
Creatinine, µmol/L	89	180	200	110	96	99	64-104
Estimated glomerular filtration rate GFR (Chronic Kidney Disease Epidemiology Collaboration), mL/min/1.73 m^2^	>60	34	32	65	75	73	—
Total calcium, mmol/L	2.41	2.57	2.32	—	—	—	2.15-2.50
Total protein, g/L	63	64	59	66	65	58	60-78
Albumin, g/L	41	47	41	47	48	42	35-52
Lactate dehydrogenase, μ kat/L	—	—	3.54	—	—	—	1.67-3.17
White blood cell count, ×10^9^/L	3.96	2.59	2.69	3.32	3.76	2.72	3.92-10.40
Hemoglobin, g/L	64	56	50	128	149	120	140-170
Mean corpuscular volume, fL	108.9	106.8	106.8	97.3	99.6	101.5	83.1-101.6
Platelet count, ×10^9^/L	81	69	70	163	204	147	171-388
β-2 Microglobulin, mg/L	—	—	29.7	—	—	—	0.8-2.2
κ serum free light chains, mg/L	7026	—	8245	1856.5	1239.8	282.7	3.3-19.4
λ serum free light chains, mg/L	9.6	—	11.9	13	7.9	14.5	5.7-26.3
κ:λ ratio	731.88	—	692.86	142.81	156.94	19.50	0.26-1.65

Two months later, the patient presented to his local clinic with ongoing severe back pain. Biochemistry showed worsening renal impairment, now qualifying as an MDE, along with worsening cytopenia, and new-onset hypercalcemia, although the latter did not reach the CRAB diagnostic threshold. The MM screening results from the recent hospital admission were noted. The clinical hematology department was consulted, and a BMAT biopsy was performed. CD138 immunohistochemical staining revealed a diffuse plasma cell infiltrate of approximately 90%, with background κ and λ staining. A sample was not sent for flow cytometry. Fluorescence in situ hybridization was negative for 17p13 deletion and 14q32 rearrangement.

At this stage, the patient was diagnosed with κ light chain MM based on the following MDEs: markedly elevated κ SFLC concentration and involved to uninvolved SFLC ratio; bone marrow plasmacytosis greater than 60%; anemia; and renal impairment, despite the absence of an M-protein spike on SPE and SIFE. Oral cyclophosphamide and dexamethasone were initiated. The patient subsequently completed 6 months of TB treatment, showed clinical improvement, and achieved full independent ambulation.

Two months after treatment initiation, a spot urine immunofixation (UIFE) revealed a small area of κ restriction, reported as a free κ M-protein (Figure 2B). At this time, the discrepancy between the initial SIFE and SLFC results was noted. The SIFE was repeated and again demonstrated κ staining in the ɑ-2 region without restriction (Figure 2C). The SIFE remained unchanged after pretreatment with β-mercaptoethanol (BME) and Fluidil (Sebia, Lisses, France) (Figure 2D). The SIFE for immunoglobulin D (IgD) and IgE heavy chain determination revealed similar κ staining with no corresponding IgD or IgE heavy chains and was also reported as negative.

**Figure 3.**
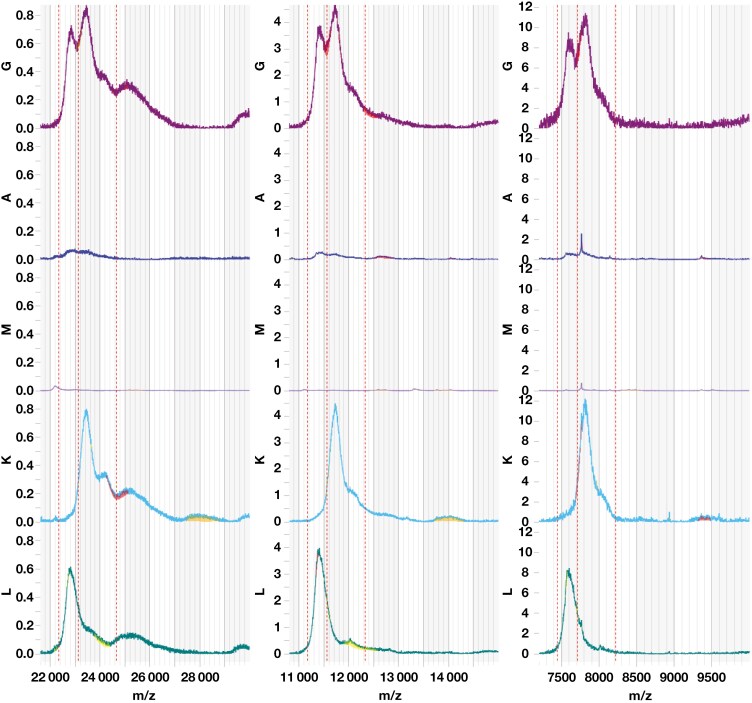
Isotype-specific light chain m/z distributions for +1. + 2 and + 3 light chains for immunoglobulin G (IgG; purple), IgA (blue), IgM (purple), κ (teal), and λ (green). All distributions are polyclonal.

Because of these discordant findings, serum and urine were referred for isotype light chain mass distribution analysis by Mass-Fix at Mayo Clinic (Rochester, MN). The light chain mass distributions did not exhibit a peak, which is consistent with polyclonal κ SFLC (Figure 3).

**Figure 4.**
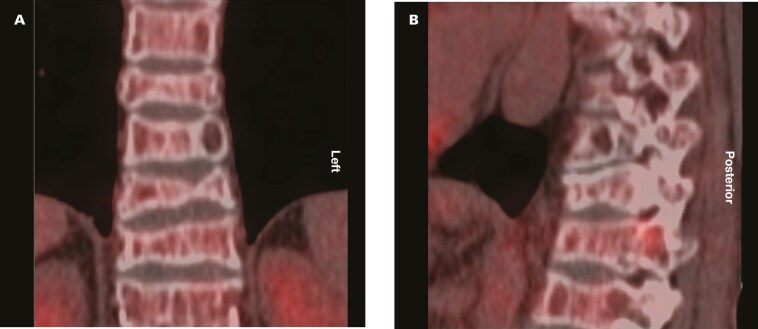
^18^F-fluorodeoxyglucose–position emission tomography/computed tomography scans demonstrating axial lytic lesions and compression fractures. (**A**) coronal view; (**B**) saggital view

**Figure 5.**
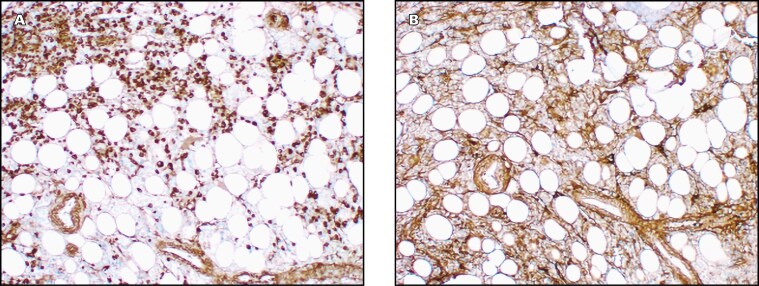
Bone marrow aspiration and trephine biopsy immunohistochemistry demonstrating (**A**) predominant κ staining compared with (**B**) λ staining (×50 objective lens).

In October 2024, an ^18^F-fluorodeoxyglucose–positron emission tomography/CT (FDG-PET/CT) revealed multiple lytic lesions involving the axial and appendicular skeleton (Figure 4). Axial skeletal lesions are illustrated in the coronal (Figure 4A) and saggital (Figure 4B) views. No lymphadenopathy was observed, and the lungs showed chronic fibrotic changes without active TB. A repeat spot UIFE showed no detectable M-protein. A repeat BMAT biopsy, after 16 months of treatment with cyclophosphamide and dexamethasone, revealed approximately 60% plasmacytosis, with predominant κ staining (Figure 5). Lenalidomide was added to the patient’s treatment regimen in November 2024, resulting in a marked reduction in κ SFLC concentration.

## Discussion

This case describes an unusual migration pattern of polyclonal κ SFLCs in the ɑ-2 region and was initially suspected to represent aberrantly migrating monoclonal-free κ, but this suspicion was not confirmed by Mass-Fix. Based on a literature search using the terms "serum free light chains", "polyclonal serum free light chains", "kappa free light chain"; "kappa:lambda ratio", "polyclonal hypergammaglobulinemia", and "multiple myeloma", we could not identify any prior case reports describing a polyclonal increase of κ free light chains with a markedly elevated κ:λ ratio. The broadened appearance may relate to multimer formation or glycosylation-related changes.^8^

The UIFE revealed a small but distinct area of κ restriction. Although this finding is consistent with a κ monoclonal process, the size of this restriction was disproportionately small relative to the markedly elevated κ SFLC concentration. This result suggests 2 concurrent processes: a monoclonal κ clone and a polyclonal proliferation of κ-producing plasma cells.

Protein migration on electrophoresis is influenced by charge, size, and shape.^9^ These properties can be altered by posttranslational modifications such as N-glycosylation, which has been implicated in malignancies, including MM.^8,10^ In addition, monoclonal SFLCs may undergo polymerization in serum, appearing not as an area of restriction on electrophoresis but rather as a “smear” representing various polymeric forms.^11^ In this case, hypogammaglobulinemia on SPE prompted a reflex SIFE because it can be a manifestation of light chain MM.^12^ The unexpected migration in the ɑ-2 region on the index SIFE, however, along with the discordant UIFE result, prompted a repeat SIFE after pretreatment with BME, which reduces disulfide bonds between immunoglobulins and converts polymers into monomers. This process typically improves immunoglobulin migration and enhances resolution on agarose gel electrophoresis.^13^ Notably, BME pretreatment did not substantially alter the migration pattern. Mass-Fix demonstrated only polyclonal κ SFLC, consistent with the broad migration detected on SIFE. In addition, the UIFE findings were atypical of a light chain MM, where an overflow pattern would typically be expected.^8^ We hypothesize that the restricted band noted on the initial UIFE was transient and that the discordance with the SFLC concentration was due to κ SFLC multimers, preventing filtration into urine. This hypothesis could also explain the absence of restriction on subsequent UIFE.

The decision to initiate treatment for MM in the patient in 2022 was based on the presence of MDEs, including renal impairment, elevated κ SFLCs with ratio greater than 100, and a BMAT biopsy showing more than 60% plasmacytosis. The monoclonal process was confirmed with bone marrow plasma cell immunohistochemistry. Although the absence of a corresponding polyclonal rise in λ remains unexplained, the use of the SFLC ratio alone should not be used as the definitive factor for the diagnosis of MM in this case.

A differential diagnosis of spinal TB (Pott disease) was considered, given the concurrent pulmonary TB at the time of MM diagnosis. This form of extrapulmonary TB accounts for approximately 2% of all TB cases and typically arises from the hematogenous or lymphatic spread of *Mycobacterium tuberculosis*.^14^ Diagnosis is suggested by characteristic imaging findings and definitively confirmed through tissue analysis, which remains the gold standard.^15^ MM can mimic spinal TB, making it an important differential in patients with spinal lesions.^16^ A key distinguishing feature is that MM typically spares the intervertebral discs, while TB often involves both the vertebral bodies and discs.^17^ In this patient, spinal x-rays showed preserved intervertebral discs. Regrettably, no histologic confirmation to exclude spinal TB was performed. Following TB treatment, however, clinical improvement was noted, constitutional symptoms were absent, and a later FDG-PET/CT scan showed no active pulmonary disease, unlike the initial CT chest findings.

MM is classified into secretory, nonsecretory, and oligosecretory subtypes based on the presence and concentration of detectable M-proteins. Approximately 95% to 97% of cases are secretory, with a detectable M-protein on SIFE or UIFE. Nonsecretory MM lacks an identifiable M-protein, while oligosecretory MM is defined by M-protein levels below the electrophoretic detection threshold of less than 1 g/L but with an abnormal involved SFLC concentration or ratio.^18^ The presence of polyclonal κ SFLCs complicated the initial diagnosis of κ light chain MM because no definitive M-protein could be identified. Lenalidomide is an immunomodulatory agent used in the treatment of MM.^19^ Following its initiation, there was a clinically significant reduction in κ SFLCs, but it remains unclear whether this rapid reduction can be attributed solely to the drug’s immunomodulatory effects. Given the absence of a discernible M-protein on SIFE, with a small κ restriction on UIFE and a polyclonal increase in κ SFLCs, the patient’s condition is currently being managed as an oligosecretory MM. In this context, treatment response will best be monitored through BMAT biopsy and PET scan.^5,20^

To the best of our knowledge, this is the first reported case of a markedly elevated polyclonal κ SFLC chain result accompanied by a clinically significantly elevated free κ:λ ratio. This case illustrates the diagnostic challenges encountered and the limited utility of SFLC and SPE in monitoring treatment response for this patient with MM.
